# Head Tracking of Auditory, Visual, and Audio-Visual Targets

**DOI:** 10.3389/fnins.2015.00493

**Published:** 2016-01-06

**Authors:** Johahn Leung, Vincent Wei, Martin Burgess, Simon Carlile

**Affiliations:** Auditory Neuroscience Laboratory, School of Medical Sciences, University of SydneySydney, NSW, Australia

**Keywords:** auditory perception, motion perception, tracking, localization

## Abstract

The ability to actively follow a moving auditory target with our heads remains unexplored even though it is a common behavioral response. Previous studies of auditory motion perception have focused on the condition where the subjects are passive. The current study examined head tracking behavior to a moving auditory target along a horizontal 100° arc in the frontal hemisphere, with velocities ranging from 20 to 110°/s. By integrating high fidelity virtual auditory space with a high-speed visual presentation we compared tracking responses of auditory targets against visual-only and audio-visual “bisensory” stimuli. Three metrics were measured—onset, RMS, and gain error. The results showed that tracking accuracy (RMS error) varied linearly with target velocity, with a significantly higher rate in audition. Also, when the target moved faster than 80°/s, onset and RMS error were significantly worst in audition the other modalities while responses in the visual and bisensory conditions were statistically identical for all metrics measured. Lastly, audio-visual facilitation was not observed when tracking bisensory targets.

## Introduction

Motion tracking is a fundamental behavior that incorporates motion processing with feedback from the sensory systems including auditory, visual, and vestibular information. Everyday examples include tracking (and avoiding) a fast moving vehicle or following and predicting the trajectory of an incoming pitch of a cricket ball (Mann et al., 2013). This is commonly associated with gaze control in vision and substantial research has examined the underlying visual-vestibular interactions (Ackerley and Barnes, 2011; Cullen, 2012). Such work has yielded important insights into a number of head motor control deficits such as cervical dystonia (Shaikh et al., 2013) and efference copy malfunction in schizophrenia (Levy et al., 2010). In the real world tracking behavior is not restricted to vision alone. In particular, moving objects are rarely silent and auditory input can be important in a multisensory context or even critical in a unisensory situation, such as tracking a fast moving car in the dark or following a wasp buzzing around our heads. Yet our understanding of this simple behavior in audition is limited.

We are aware of only two studies: Beitel (1999) and Scarpaci (2006), that have examined head tracking of moving sound. Beitel (1999) studied the dynamics of auditory tracking in cats by recording their head motion when tracking a series of clicks emitted by a speaker rotating at 12 or 16°/s. Using cats with optical nerves that were sectioned to eliminate visual involvement, the cats reacted to moving sounds in two phases: (1) a rapid head orienting response to localize the target followed by a (2) a maintenance phase that consisted of a succession of stepwise movements involving cycles of overshoot-and-pause, which ensured the target was close to the midline. This response has a passing resemblance to the visual pursuit of acoustical targets and is suggestive of a series of stepwise localization task. Scarpaci (2006) examined the head tracking accuracy of auditory stimuli in humans as a means to verify the accuracy of a real-time virtual auditory space (VAS) rendering system. The subjects were asked to track a band-limited stimulus filtered with non-individualized head related transfer functions (HRTF) that moved around the head in a pseudo-random manner. The time lag of the head tracker was varied, demonstrating that tracking error increased as a function of head tracker delay. These two studies provided glimpses into auditory tracking behavior and highlighted various methodological challenges. However, to understand the underlying sensorimotor feedback mechanisms, there needs to be a clearer picture of the behavioral norms and biological constraints involved in auditory tracking.

In this study, we systematically examined auditory head tracking over a wide range of stimulus velocities. By combining individualized VAS and real time head tracking, we rendered realistic auditory targets that were perceived to be moving externally around the subject (source motion), while creating a cohesive auditory space by constantly monitoring and correcting for subjects' own head movements (self motion). Unlike vision, there is a lack of evidence for the existence of low-level auditory motion detectors in audition and the prevailing notion is that a form of “snapshot” processing facilitates motion perception (Grantham, 1997; Carlile and Leung, accepted). This suggests that in a tracking task subjects can compare the positional differences between head and target locations in each “snapshot” window and correct their trajectories accordingly. It is uncertain, however, if “binaural sluggishness” that is inherent in auditory spatial processing, may limit that rate at which subjects can accurately track a moving target (Grantham and Wightman, 1978). Likewise, the biomechanics of head movement may impose a ceiling on the velocity at which subjects can accurately control their head rotations. Also, of interest is whether subjects' performance will differ between audition and vision tracking, given the differences in mechanisms underlying motion processing. As such, we will compare the auditory responses to a control condition that asked the subjects to track a moving visual target in the dark at identical speeds and trajectories. Lastly, by integrating the auditory and visual presentation systems, we were able to examine a “bisensory” condition using spatially aligned auditory and visual targets. Previous work has shown that cross modal interactions can affect audio-visual motion perception even though the spatial acuity of vision is superior to audition (Wuerger et al., 2010; Schmiedchen et al., 2012).

## Materials and methods

### Subjects

Six volunteers (two females and four males, ages 24–50) participated in this study. This cohort was drawn from within the University of Sydney student pool, and with the exception of one subject, were naïve to the task. All subjects had normal or corrected vision and normal hearing as tested under clinical audiometry (up to 8 kHz); furthermore, none of the subjects reported previous history of cervical dystonia, related neurological deficits, difficulties in head movements or neck stiffness. All participants provided written consent in accordance with ethics requirements approved by the University of Sydney Ethics Committee.

### System description

The tracking system recorded the subject's head motion in near real-time as they tracked a moving auditory, visual or audio-visual object by pointing their nose. The system was based on software written in Mathworks Matlab 2009b and 2013a running on a Windows PC (Xeon Quad core) that integrated Intersense IC3 and IC4 head trackers, RME Fireface 400 audio interface and a programmable LED array for visual display (see component descriptions below). System latency was minimized by delegating essential operations to the hardware components. Software interfaces that were written and compiled to ensure minimum latencies were also used. The system used the native system timing commands to achieve an average of 2 ms resolution. The maximum system latency to execute each program cycle was 2.5 ms (see Section Auditory Stimulus). The head tracker had a maximum update rate of 180 Hz and rated to angular speeds of 1200°/s.

### Auditory stimulus

#### VAS generation

Individualized VAS was used to create the moving auditory stimuli as this has numerous advantages over traditional methods such as movable speakers and stereo balancing with speaker arrays. It generates no mechanical noise when moving, can be moved at speeds of over 100°/s and produces no acoustical transients on activation. Instead, it provides a high degree of flexibility in setting the parameters of motion, with fine-grained control over path, velocity and acceleration. Here, broadband white noises were filtered with the subject's HRTFs that were recorded at 1° spatial intervals (see below). To ensure a smooth transition between positions, the post-conditions of the previous filtering output stage were interpolated with the pre-conditions of the next stage. Traditionally, VAS delivered over headphones has a head centered frame of reference, where the locations of the stimulus shift in accordance with head position. In order to decouple the auditory (“source”) frame from the head (“self”) frame of reference, the system computes the difference between the actual sound and current head location (based on the head tracker output). A stimulus can then be generated that accounts for the orientation of the head. In practice, by regularly monitoring the head position and adjusting the location of the target to compensate for any movement, a perceptually static source can be produced. For a sound that is moving, provided that the subject maintained perfect head tracking of the source location, the sound will maintain a fixed spatial location in front of the subject's head. In this experiment, velocity was manipulated based on the duration of sound (in milliseconds) per degree of movement. In this context, it is essential that a precise sampling resolution be maintained otherwise a “slippage error” will occur, where the stimulus position is corrected erroneously by a delayed head position sample. Timing measurements of the core stimulus generation code showed an average execution time of 2 ± 0.5 ms for each cycle consisting of the following main steps: (1) head position sampling from the head tracker, (2) frame of reference correction, and (3) HRTF filtering and delivery. Each cycle was delimited by the length of the stimulus at each position (e.g., a 100°/s moving stimulus will have a 10 ms time cycle and a 50°/s stimulus will have a 20 ms cycle). While this may present a situation where sampling time increased when stimulus velocities decreased, care was taken to ensure that the sampling resolution is above sensory threshold. Previous work by Brungart et al. (2006) established that head tracking latency in excess of 73 ms will lead to a decrease in localization accuracy for static targets and that a 30 ms latency is perceptually irrelevant. As such, for velocities slower than 50°/s the system subsample space by halving the sampling time; for example, with the lowest stimulus velocities of 20°/s, the sampling time will be 25 ms rather than 50 ms. Subjects were asked qualitatively about their perception of the stimulus: (1) whether the targets were externalized outside their heads, (2) were there any apparent change in sound quality such as jitter and jumps during source and self motion. All subjects reported that the tracking and presentation system rendered a smooth and externalized auditory space.

#### HRTF recordings

HRTFs were recorded individually for each subject using a “blocked-ear” recording technique (Møller et al., 1995). Subjects' ear canals (outer portion) were sealed with a mold made with dental extrusion gel that was used to hold the recording microphone (Knowles FG23329). Then subjects were seated in an anechoic chamber of size 64 m^∧^3 with a 99% sound absorption above 300 Hz. Inside the anechoic chamber, a semicircular robotic armature system can move a speaker (Audience A3, apex mounted) to any location in space (above −40° elevation) 1 m away from the participants head (described detail in Carlile et al., 1997). Prior to the recording the subject's head was aligned with two lasers mounted on the robotic arm. A single pole coordinate system describes space, where the right hemisphere goes from 0 to 180° Az and positive elevations describe positions above the audio-visual horizon. HRTFs were recorded at 1° intervals along the audio-visual horizon using a 1 s exponential sine sweep (Fontana and Farina, 2006). In order to reduce the recording artifacts from inadvertent head movements, a head tracker (Intersense IC3) was used to continuously monitor the subject's head position. The automated recording procedure paused whenever head motion was detected. This system was controlled by a Windows PC running Matlab 2009b with a RME Fireface 400 audio interface.

#### Playback

A pair of Sennheiser HD650 open-back circumaural headphones were used for VAS playback and its transfer function was also recorded for each subject in the same anechoic environment. Five repeat headphone calibration recordings were made where the subjects were asked to remove and re-seat the headphones. The average of the five recordings was taken as the calibration recording (Pralong and Carlile, 1996). Prior to stimulus generation, the calibration recordings of the microphone and headphones were removed from the HRTF recording using the Kirkeby inverse (Fontana and Farina, 2006). The fidelity of the individual's recording was verified via a series of virtual space localization experiments (see Section Results).

### Visual stimulus

Apparent visual motion was generated using a high density LED strip containing 100 equally spaced red LEDs mounted on a semicircular wooden frame of 1 m radius. Each of the LEDs was individually controlled via a WS2801 integrated controller. This LED strip was connected to the tracking system via an Arduino Mega2560 platform and a custom Matlab software interface. By pulsing each LED sequentially, apparent visual motion was created appearing as a short line segment moving in the direction of motion. In this experiment, the velocity of motion was controlled by varying the on-off time of each LED. To ensure the correct velocity was attained, the system was calibrated using two photo diodes placed at various locations along the path. By measuring the time difference between the excitation of the diodes we were able to check the actual stimulus velocity. Output from these photo diodes was recorded and measured using a digital oscilloscope and also sampled via an analog-digital converter. Repeated measurements were made at different locations under various velocities and the deviations were within 1°/s.

### Audio-visual “bisensory” stimuli

A stimulus containing both moving audio and visual components was created by presenting the moving sound and apparent visual motion in synchrony (Sankaran et al., 2014). Particular care was taken to ensure accurate spatio-temporal synchrony between the two modalities by calibrating and comparing the output of the photo-diodes (see above) with an auditory calibration stimulus at each velocity. A number of calibration positions were taken. Photo diodes were placed at these positions on the LED array. In audition, pure tone pips of 10 ms were embedded in the broadband noise, at temporal offsets that corresponded to these calibrating positions. The output of the photo-diodes and auditory stimulus were looped back into the RME Fireface interface to ensure that no samples were dropped in the recording process. By comparing the activation time of the photo-diodes with the position of the 10 ms tone pips, we were able to synchronize the auditory and visual stimuli.

### Experiments

#### Localization validation

The fidelity of the HRTF recordings was validated by a series of localization training and test sessions under free field and VAS conditions (Jin et al., 2004). In the free field, subjects were given a series of training sessions where they were asked to point their noses toward a static 150 ms noise source (broadband white noise) positioned randomly in space. Auditory and visual feedback was provided and subjects were reminded to use their noses rather than eyes for pointing to the perceived location. After the subject gained proficiency in the task, localization accuracy was tested in a series of 5 localization sessions that provided no feedback. Each session consisted of 76 positions conducted inside the anechoic chamber in the dark.

In the VAS condition, sounds were generated by filtering a broadband noise burst with the individual's HRTF filters and presented over headphones. The set of locations presented were chosen from the tracking experiments at ±50°, ±35°, ±20°, ±10°, ±5, ±2, and 0° Az along the audio-visual horizon. Two forms of localization tests were performed: (1) static short burst noise (head fixed) and, (2) sustained sounds with head movement (head free). In the head fixed condition, a 150 ms noise burst was presented while subjects' head remained fixed in front (as recorded by the head tracker), this is identical to the free field training and testing condition. In the head free condition, a 3 s noise was presented during which the subjects were free to move their heads. Since the tracking system continuously compensates for subjects' head movement, subjects' perceived the target as fixed at the actual location in space.

#### Motion target tracking

**Figure 1:**
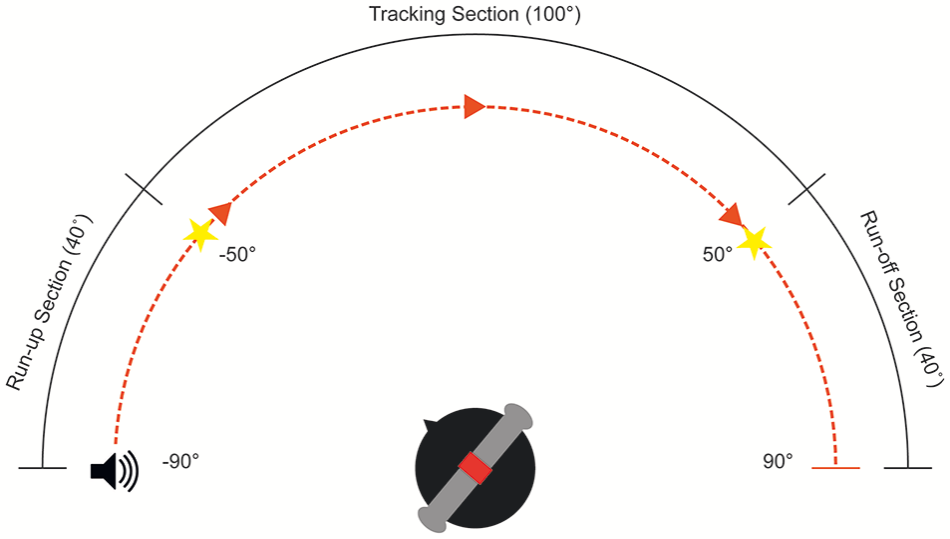
**Schematic diagram of the head tracking experimental setup**. Subjects fixate toward an LED located at either ±50°Az, depending on direction of motion. The target will begin moving at ±90°Az, providing a 40° run-up section.

The aim of this experiment was to examine the ability of subjects to track a moving stimulus with their heads between ±50° Az (frontal hemisphere, audio-visual horizon) at speeds from 20 to 110°/s at 10°/s intervals for both left and rightward moving objects. This 100° maximizes the tracking radius while maintaining a comfortable neck turn range (see Figure 1) Subjects were seated in the center of a light-attenuated dark room, and their initial position was calibrated using two guiding lasers. Ten training trials were presented in each session to familiarize the subjects with the task and stimulus. Subjects began by fixating to one of the two LEDs at +50 or −50°, this maximizes the tracking radius while maintaining a suitable neck turn range (see Figure 1). The stimulus onset started at ±90°, giving a 40° “run-up” arc where the subjects were asked to keep their head stationary. This provided the subjects with the opportunity to estimate the velocity of the stimulus. They were instructed to start tracking only when the stimulus reached the location of the fixation light, and ensure that their nose was pointing to the stimulus at all times during the trial. The fixation light at the tracking end point was lit up as an indication that the trial was complete.

Three separate experiments examined auditory, visual, and auditory-visual tracking performance. For each experiment, the 10 velocities were presented randomly with 10 repeats for each velocity and direction totaling 200 trials. These were divided into four blocks of 50 trials. The direction of motion alternated each trial. A single block took approximately 5 min to complete and a short break was provided between blocks to avoid fatigue.

### Data analysis

All statistical analysis was conducted using a combination of Matlab (Mathworks) and Prism (GraphPad) software. Unless otherwise stated, ANOVA with multiple comparisons were Tukey corrected and confidence intervals of the group means were derived from a non-parametric bootstrap with replacement (*N* = 1000).

The three metrics analyzed were onset error, RMS error, and gain. These were calculated from a subject's head movement trace, from the point of head movement onset to the end of tracking (see **Figure 4**). The onset position was estimated using the “knee point” in the head movement traces. This knee point was calculated using a bisected linear fit that minimized the fitting error of two line segments. Simply, a bisection point was arbitrarily defined (near the beginning of the head tracking trace), and then sequentially moved along the trace. At each increment two line segments were fitted and the knee point was the bisection point that minimized the sum of errors of the fits. *Post-hoc* visual checks of the analysis showed that this method was robust and incorporated the head-resting tremor during fixation.

## Results

### Localization control

**Figure 2:**
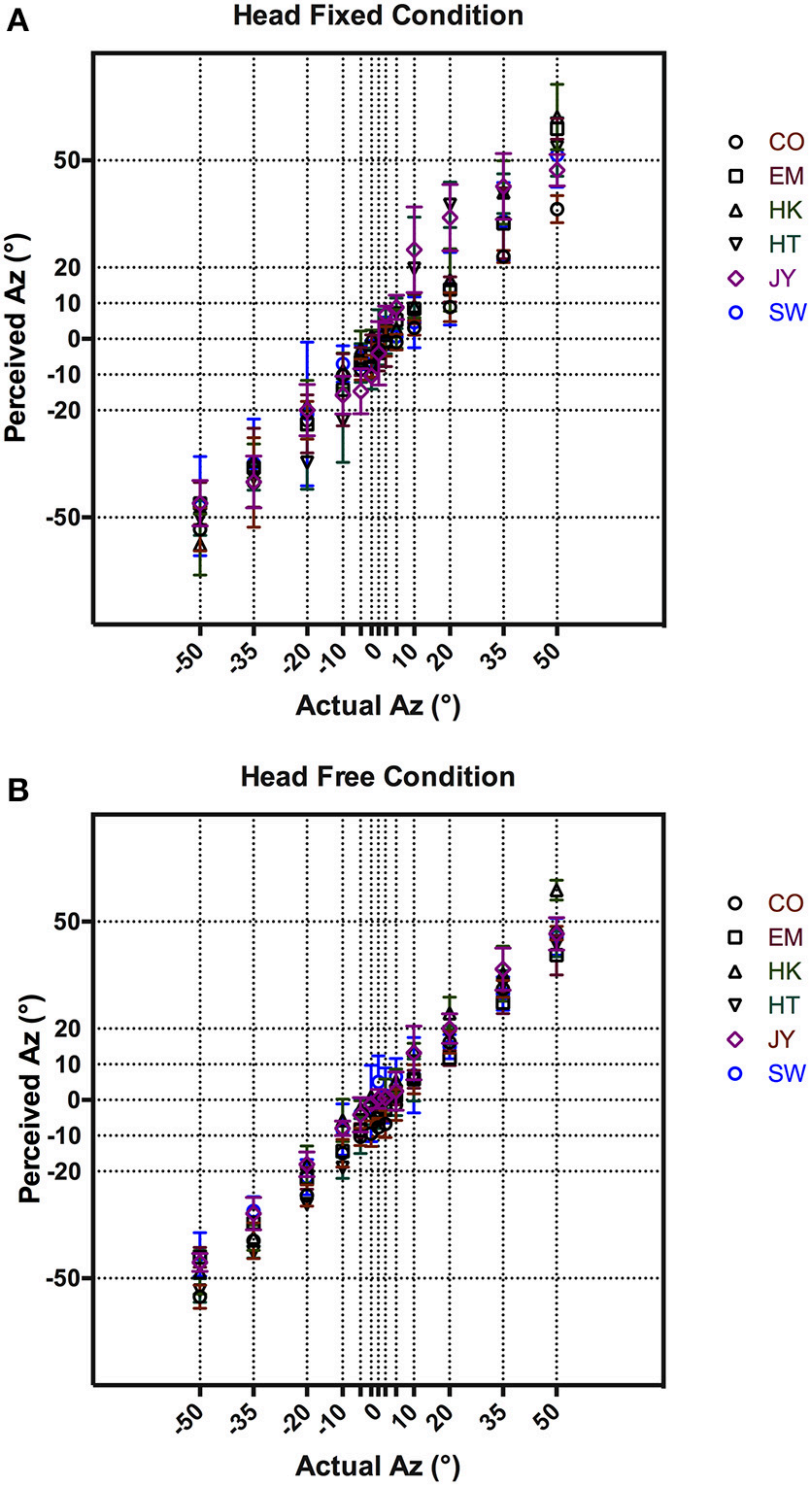
**VAS localization results comparing between perceived and actual azimuth in head fixed (A) and head free (B) conditions**. Mean responses from all subjects are shown with error bars representing 95% CI.

Figure 2 shows each subject's responses of actual vs. perceived azimuth averaged across five repeat measures. The results showed a tight distribution of responses in the frontal region (–5 to 5°) under both head fixed (Figure 2A) and head free (Figure 2B) conditions. As expected, localization accuracy decreased in the head fixed condition as the target azimuth moved further to the sides, as illustrated by the increased variances in Figure 2B at target azimuths >±20°. This was not observed in the head free condition (Figure 2B). Overall, subjects performed consistently and accurately. Pooled across subjects and the 13 target azimuths, the average localization error was 4.6° with STD of ±1.8 and 3.9° with STD of ±1.4° for head fixed (Figure 2B) and head free (Figure 2B) conditions, respectively.

### Tracking analysis

**Figure 3:**
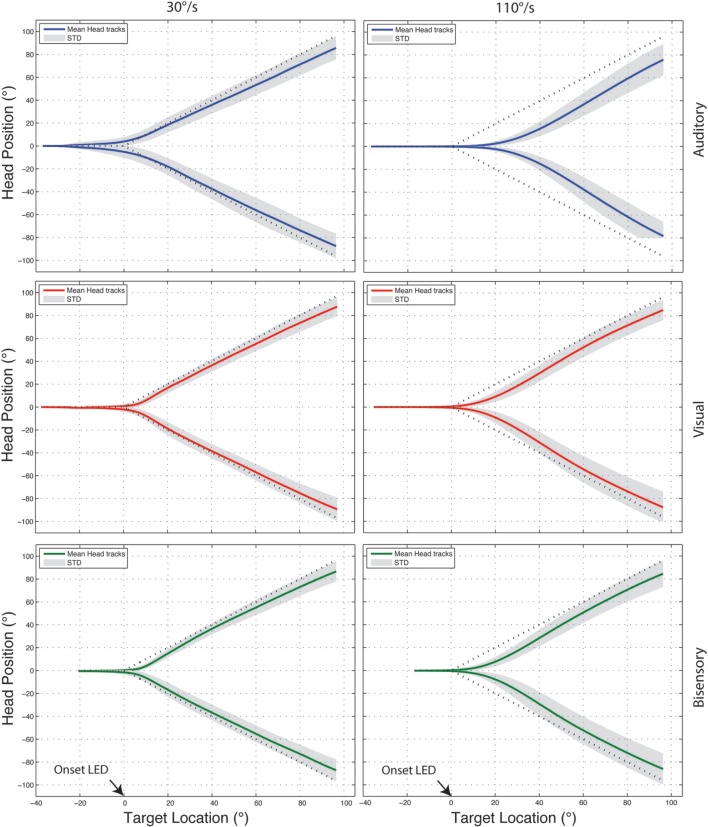
**Example head tracking traces for one subject tracking targets moving at 30 and 110°/s**. Positive y-axis values correspond to rightward head motion and time is plotted on the X-axis. In all cases the head position averaged across 10 trials is shown with the standard deviation shaded in gray and the dotted line marking the ideal response. All three targets modalities are shown—auditory (blue), visual (red), bisensory (green).

The fast sampling rate and high spatial sensitivity of the recording system generated a highly redundant data set for each trial, as such, before data analysis, the “raw” recorded traces were quantized to 0.5° (from 0.03°, the head tracker resolution). Figure 3 shows examples of the quantized head tracking responses for auditory (blue), visual (red), and bisensory stimuli (green), for one subject comparing between a slow (30°/s) and fast velocity (110°/s). Qualitatively at 30°/s, tracking responses all followed the general shape of the ideal curve with insubstantial differences between the three stimulus conditions. At 110°/s however, there was an increasing lag in head movement initiation that lead to substantial “under turn” from the ideal response that was most evident under the auditory only condition. Three metrics were used to quantify this tracking behavior: (1) onset error, (2) RMS error, and (3) gain. All results shown combined the leftwards and rightwards motion, pooled across the six subjects.

**Figure 4:**
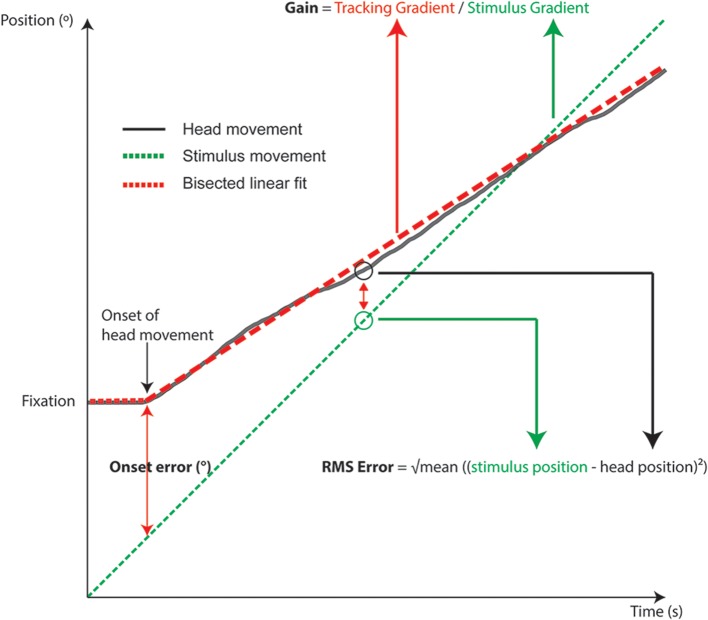
**Schematic diagram of an individual head tracking trace overlaid with target motion, showing how gain, RMS, and onset errors are calculated**.

#### Onset

**Figure 5:**
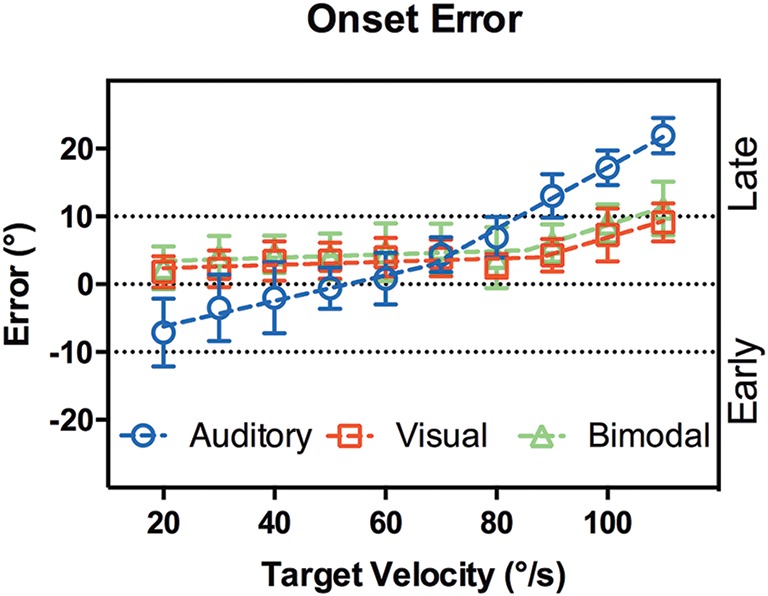
**Onset error vs. target velocity**. X-axis shows the target velocity, Y-axis shows the onset error averaged across the subjects. Error bars denote 95% CI. Dotted lines are the lines of best fit. In audition (blue circles), a segmented linear regression was preferred over a simple linear fit, whereas the converse was true for vision (red squares) and bisensory (green triangles). When the actual target lags behind head movement, onset error will be less than 0°, marked as the “Early” region.

Figure 5 shows the onset error vs. target velocity, defined as the target position relative to head movement onset, with positive values indicating a lag in head onset. It is clear that visual and bisensory responses followed the same trend. A comparison of nonlinear regression models confirmed the null hypothesis that results from both modalities can be accounted for by a single model [*p* = 0.166, *F*_(4, 112)_ = 1.7]. Importantly, a comparison between simple linear and segmented regression rejected a simple linear fit, instead favoring a two segment model [*p* = 0.007, *F*_(2, 56)_ = 5.5] with an inflection point at 87°/s (95% CI [77,90], *R*^2^ = 0.91). The line segments were:

$$\begin{array}{lll} y\; & = & \;0.024x\;+\;2.4,\text{ for }x\;<\;87^{{}^{\circ}}∕\text{s},\text{and}\\ y\; & = & \;0.25x\;+\;4.5,\text{ for }x>\;87^{{}^{\circ}}∕\text{s} \end{array}$$

While the mean onset error between target speeds of 20 to 80°/s was 3.08 ± 0.83°.

However, performance in the auditory condition differed significantly when compared to visual and bisensory conditions [*p* < 0.001, *F*_(4, 112)_ = 42]. While a 2 segment linear fit was still preferred, the inflection point was significantly slower at 68°/s (95% CI [55,82], *R*^2^ = 0.98). The line segments were:

$$\begin{array}{lll} y\; & = & \;0.19x\;-\;10,\text{ for }x\;<68^{{}^{\circ}}∕\text{s},\text{and}\\ y\; & = & \;0.45x\;+\;2.9,\text{ for }x\;>\;68^{{}^{\circ}}∕\text{s} \end{array}$$

This showed that when tracking the slower moving sounds (<60°/s) there was a tendency for subjects to move their heads too early—before the target even arrived at the fixation point. Further, a multiple comparison analysis showed that between 60 and 80°/s, there were no significant differences in onset error between the modalities. For target velocities outside this range, auditory performance significantly decreased (see Supplementary Table 1).

#### RMS error

RMS error was calculated by a point-by-point comparison of the target position against the subject's head position using the following equation:

$$\begin{array}{l} RMS\;error\;=\;\sqrt{mean{\left(Head\;locations\;-\;Target\;locations\right)}^{2}} \end{array}$$

(The 40° “run up” arc of the target movement was excluded from the calculation).

**Figure 6:**
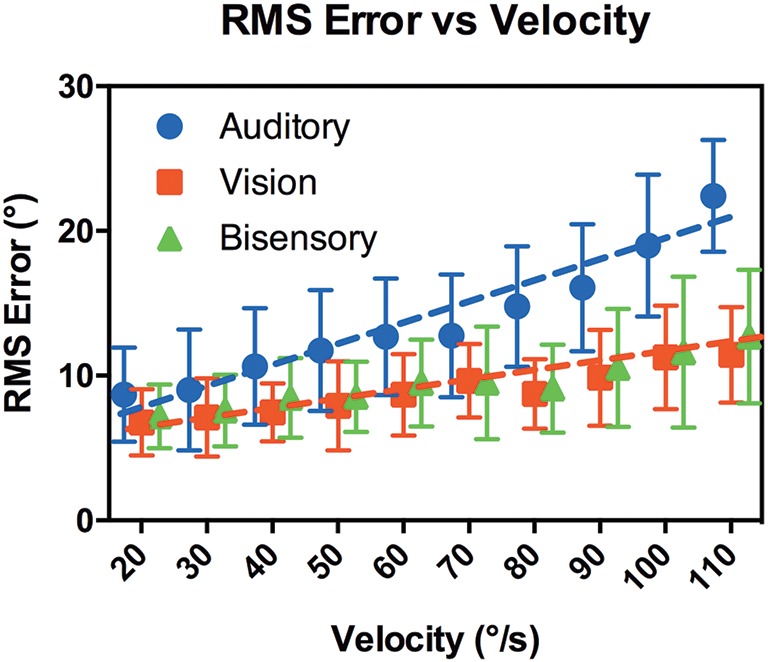
**RMS error vs. target velocity**. X-axis shows the target velocity, Y-axis shows the RMS error averaged across the subjects for each modality. Error bars denote 95% CI. The dotted lines are the lines of best fit.

Figure 6 compares RMS error against target speeds. A comparison of nonlinear regression models again showed that visual and bisensory results can be represented by the same line fit [*p* = 0.54, *F*_(2, 116)_ = 0.63, *R*^2^ = 0.88] of: *y* = 0.053*x* + 5.7. Whereas, the nonlinear regression model for audition was significantly steeper [*p* < 0.001, *F*_(2, 116)_ = 43, *R*^2^ = 0.93] with a fit of: *y* = 0.14*x* + 4.7. A multiple comparison analysis between modalities and velocities highlighted that RMS error did not differ substantially between modalities from 20 to 70°/s, but diverged for the faster speeds in auditory tracking (see Supplementary Table 2). At 110°/s, there were highly significant differences between audition and the other modalities, with mean difference of 11.0, 95% CI [5.82, 16.1] (Auditory vs. Visual, *p* < 0.001) and 9.72, 95% CI [4.56, 14.9] (Auditory vs. Bisensory, *p* < 0.001).

#### Gain

**Figure 7:**
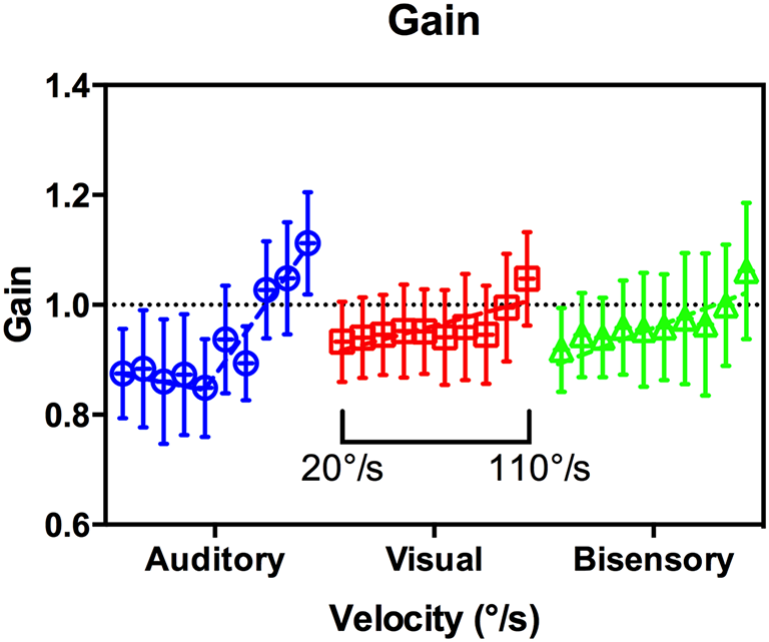
**Gain vs. target velocity**. Grouped by modality for clarity, velocity ranged from 20 to 110°/s within each subgroup as indicated. A segmented linear regression was preferred in Audition (blue circles), whereas a simple line fit was preferred for Vision (red squares), and Bisensory (green triangles).

In this analysis, gain is defined as a metric that describes whether subjects were able to correctly match the speed of the target. Gain is the ratio of head velocity to target velocity. This is done by fitting a line of best fit to the head position data and dividing its gradient with that of the stimulus (the stimulus velocity). It is calculated from the onset of head motion to the end of the tracking interval. The mean gain values pooled across subjects are shown in Figure 7, with a gain of 1 indicating a perfect match of velocity. There were large variances in conditions and modalities, and a multiple comparison analysis revealed no significant differences in gain between modalities across target velocities (Supplementary Table 3). However, the overall trend again highlights the differences between audition and the other modalities. In audition, a segmental linear regression with an inflection point at 61°/s (95% CI [43,78], *R*^2^ = 0.91) was significantly better at representing the data than that of a straight line [*p* < 0.006, *F*_(2, 56)_ = 5.62], whereas the converse is true in vision and bisensory.

## Discussion

To our knowledge this is the first systematic study of head tracking response to a moving auditory target in humans, testing the condition where the source (target) and self (head) are moving simultaneously at a range of stimulus velocities. While this commonly occurs in a natural listening environment, studies thus far have examined these frames of references separately—either using source motion to examine auditory motion perception, or self motion to probe auditory spatial perception (see Carlile and Leung, accepted for a review). As such, the complex sensorimotor interaction in a dynamic environment remains unclear. Beitel (1999) examined the acoustic tracking responses in cats using a slow moving target that subtended a 36° arc in free field. Two response phases were characterized: onset and maintenance. The onset phase comprised of a head movement toward the target based on its initial velocity and direction. This onset response closed the gap between the head and target to lead into the maintenance phase of tracking, characterized by a succession of small stepwise head movements about the stimulus location. Here, we examined human responses with a wider range of velocities (20–110°/s), trajectory (100°) and modalities (audio, visual, and bisensory), also characterizing the tracking profiles into onset and maintenance phase. Our results showed consistent patterns that can help delineate the complexities of the underlying sensorimotor feedback loop (see below), but we also observed individual differences and within subject variations. One source of variability can be attributed to the biomechanics of the head movement. Unlike eye movements, the group of muscles responsible for head motion have a degree of redundancy and are not aligned into push-pull pairs (Beitel, 1999; Peterson, 2004). It has been observed that different muscle combinations have been used in tracking and are highly task dependent (Peterson, 2004). Even though our subjects had training and experience prior to testing it is possible that their strategies varied subtly between trials. As noted in Beitel (1999), eye-gaze interactions may also affect auditory tracking responses. While the optic nerves of the cats were resected in that experiment, we were also interested in cross modal effects so chose not to restrict visual input to just the fixation lights. Instead, our experiments were conducted in a darkened room with the only visual references being the onset LEDs.

### Stimulus validation

As discussed in Methods, our stimulus presentation system used individualized HRTFs that were recorded at 1° spatial intervals around the subject's audio-visual horizon. This was necessary to render high fidelity VAS given that subjects' head movements could be random. The resultant VAS was psychophysically validated with a series of control localization experiments in a head-fixed condition where subjects' head had to remain stationary during stimulus presentation. Further, we also tested the fidelity of the presentation system using a head free condition, where subjects were encouraged to move their heads during stimulus presentation. Subjects uniformly reported that the auditory target were clearly localizable and externalized in both conditions. As shown in Figure 2B, localization error in the head-fixed condition were tightly distributed along the midline at 0° Az and diverged from 10° Az. This is consistent with free field localization results in our laboratory as well as previous studies that showed increasing localization error from the midline (Carlile et al., 1997; Lewald et al., 2000). Carlile et al. (1997) suggested this increase in location error with increasing distance from midline could be partly due to the motor error of nose pointing as more experienced subjects appeared to have smaller localization errors. This was absent in the head-free condition (Figure 2B), where subjects' performance was largely uniform with substantially reduced variance. Given the additional binaural cues available during head movement this was not surprising, as subjects were able to fine-tune their responses by adjusting for errors otherwise made by the initial nose pointing (Thurlow et al., 1967; Wightman and Kistler, 1994). In addition, the small mean localization error and tight variance (4.53 ± 0.37° pooled across all subjects) confirmed the fidelity of VAS used in subsequent tracking tasks.

### Onset phase

We measured the spatial difference between the stimulus reaching the start location and onset of head movement (Figure 4). Apart from reflecting the time required for motor planning, the onset error is also conflated with the subject's estimation of the arrival time of the target to the onset LED, since subjects were asked to only move their heads when the target arrived at the onset position. In audition, a segmented linear analysis showed that the onset delay did not follow a simple linear trend but rather a two segment line fit was preferred, with an inflection point at 68°/s (Figure 4). For targets moving slower than 60°/s, subjects tended to begin rotation before the target reached the onset point, by 4–8°. Such a “representation momentum” effect—where the end point of a moving target is mislocalized toward the direction of motion, has been reported previously (Feinkohl et al., 2014). For velocities >60°/s, the error increased and a delayed onset was evident, by up to 22° at 110°/s. This may be due to perceptual errors in estimating the target location at faster velocities as well as the speed of the subject's sensorimotor feedback loop. In vision, where the localization accuracy is far more precise, there was no indication of an early bias in arrival estimation; rather, there was a slight delay in onset responses at the slower target velocities that was nearly constant (gradient = 0.024) until an inflection point at 87°/s. For example, when the visual target was moving at 20°/s, the subjects was behind the target by 1.8° ± 2.2° (SD) at onset compared to 2.7° ± 1.8°(SD) behind at 80°/s. This pattern was minor yet consistent and may reflect small eye-gaze discrepancies at fixation; while we were unable to track subjects' eye position, the experiment was performed in the dark to ensure adequate fixation and minimize eye movements. Given the highly localizable nature of the visual target, it was not surprising that there was a lack of early bias in onset estimation. For the faster velocities post inflection, the trend was similar to auditory tracking in that the onset error increased with velocity, but the magnitude was substantially smaller: 9.1° ± 2.7° when tracking a 110°/s visual target. While we did not systematically probe the nature of this error, it is likely driven by reaction time necessary for head onset given the mass of the head and the number of muscles involved in its engagement. Comparing visual and auditory modalities it appears that the increased onset error in audition can be attributed to a delay in resolving spatial locale in the sensorimotor feedback loop, possible due to the binaural sluggishness in the auditory system (Grantham and Wightman, 1978).

In the bisensory condition, given the accuracy and precision in visual localization we expected subjects' responses to predominantly follow what we observed in vision. A comparison of fitting parameters showed that this was true, where one curve satisfied both conditions [*p* = 0.166, *F*_(DFn, DfD)_ = 1.654 (4, 112)]. We were also interested in whether a stimulus containing spatially congruent auditory and visual components improved subject responses. Such multisensory cross modal facilitation can be modeled based on a maximum likelihood integrator and has been demonstrated for static (Alais and Burr, 2004) and dynamic (Wuerger et al., 2010) auditory-visual stimuli. If the auditory and visual spatial information were combined following that of a maximum likelihood integration, the response variances in the bisensory condition would be smaller than either of the unimodal conditions (Ernst and Banks, 2002). However, no such evidence was found in our results. It is unclear why cross modal facilitation was not observed in our experiment. It is possible that the response to our task may not have been sufficiently sensitive to detect such facilitation. This may be exacerbated by the difference in mode of stimulus presentation. In previous experiments all stimuli were presented in free field; whereas here, the visual target was in “free field” while the auditory target was in virtual space thus leading to a degree of sensory dissonance.

### Maintenance phase

In the maintenance phase subjects were expected to constantly compare their head position against the target to minimize spatial discrepancies. Our daily experience and numerous studies (Cooper et al., 2008; Leung et al., 2008) showed that our heads can move freely in a wide range of velocities, mostly as an orienting response. However, a tracking task imposes the extra requirement of constant sensorimotor feedback to compare between head position and target location, which given the variability in the biomechanics of the head may impact subjects' response profile. Here, we are interested in how accuracy varied with target velocity. Recall that targets moved in a straightforward manner without any random path changes and subjects had prior knowledge of the direction and velocity gained from the onset phase, plus a rich set of localization cues from individualized HRTFs. We expected that subjects would take full advantage of these available cues—space, time, and velocity, to accurately predict and locate the target position (i.e., minimize error) along its path at any given moment. When the targets are moving slowly, subjects should be able to freely move their heads in line with a moving target to maintain accuracy. However, at higher speeds subjects may have difficulty performing similar head movements as this requires an even faster rate of motion and acceleration than the target motion. In these cases, it is possible that subjects will follow the target by matching and maintaining the target velocity instead. We will explore these predictions below by examining the RMS error and gain function.

Figure 6 shows the RMS error averaged across subjects for the range of velocities tested. While there were individual variations the trends discussed here are consistent across subjects. Overall RMS error increased with a highly significant interaction with velocity in all modalities (*p* < 0.001). At 20°/s, we observed only a slight increase in error when compared against localization of a static target in the control cases. There, localization error was on average 3.9° ± 1.4° in the dynamic (head free) condition while RMS error in audition at 20°/s was 8.7° ± 1.2. This small increase was not surprising given that RMS error was averaged across the tracked path from the onset position and the differences in task requirement. Subjects all reported that the task was easy and an examination of the subjects' head profiles showed that most subjects were able to follow the actual location of the target sound, while some swept their heads across the target to pinpoint its exact location. Again, visual and bisensory performances were not statistically different but performance was significantly worse in audition, with a significantly steeper gradient (0.14 ± 0.027 degrees per °/s in audition, vs. 0.053 ± 0.009 degrees per °/s in the other modalities, *p* < 0.001). This suggests that all subjects could follow the targets at the slower velocities but performance deteriorated at faster velocities in audition. A multiple comparison analysis showed that differences in RMS error between audition and the other modalities became significant when target velocities reached 80°/s. We hypothesize that the overall worsening performance in audition is related to the computational nature of the binaural system that is less precise and inherently “sluggish” (see Carlile and Leung, accepted for a review) compared to the spatiotopic nature of the visual system. Previous work examining binaural integration of moving sounds have suggested subjects can only follow slow moving targets, with the minimal audible movement angle of around 5° at 15°/s that increased to more than 20° at 90°/s target velocity (Grantham, 1986). Even though these previous studies were limited to source motion where subjects' head remained stationary, the trends are comparable. At the faster velocities, the increase in RMS error in audition may in part be due to difficulties in catching up to the target after onset, as hinted at by the increase in onset error mentioned previously. It should be noted that subjects reported no difficulties moving their heads at the faster velocities, and as reported in Leung et al. (2008) even faster head turn speeds are possible. As such, it is interesting that they did not exhibit any over compensatory behavior in head turn speeds, given the *a priori* velocity information provided by the run-up period.

Gain response was the second metric analyzed during the maintenance phase, comparing the actual target velocity against the average head tracking velocity. A gain of one indicates perfect velocity matching. As part of our experimental design, the initial 40° segment was designated a “run-up,” whereby subjects had to fixate and listen to the target motion without physically moving their heads. This provided subjects with the relevant perceptual information to form an internal prior of the direction and velocity of the moving target. During pilot studies, subjects were tested without this segment and had substantial difficulties in tracking even at moderate speeds. In audition, we found that for velocities slower than about 80°/s the gain is below 1, suggesting that subjects' overall head turn speed was slower than the target speed. This was consistent with the observations of Beitel (1999) in cats. From the onset phase analysis we observed that subjects tended to engage in head motion before the target reached the onset point. Taken together, this suggests that subjects were deliberately retarding their head motion to compensate for the early onset. For targets moving faster than 80°/s the gain was greater than 1. There were substantially less deviation in the other modalities, with gain close to unity for velocities up to 90°/s. Together, the analysis during the maintenance phase suggests that subjects were able to actively compensate for the early or late onset responses.

In summary, this study examined the ability of subjects to track moving auditory, visual, and bisensory stimuli. The overall findings suggest that subjects were able to track moving auditory targets at velocities below 80°/s. The fact that performance was comparatively worst than vision and audio-vision was likely due to differences in localization precision and binaural sluggishness, leading to significant tracking errors at the faster velocities. Cross modal facilitation between auditory and visual stimulus was not observed and tracking behavior to bisensory targets was not significantly different to that of unimodal visual responses. These results describe behavioral responses to a straightforward tracking task in a simple environment, forming the basis for future research. Recent technological developments such as the Oculus Rift will allow us to explore more complex and naturalistic situations that can include multiple moving targets and unpredictable trajectories, providing important insights into human sensorimotor pathways.

## Author contributions

JL and SC conceived and designed the original experiment. VW and MB conducted the experiments. JL, VW, and MB analyzed the data. JL, VW, MB, SC all participated in preparing the manuscript.

### Conflict of interest statement

The authors declare that the research was conducted in the absence of any commercial or financial relationships that could be construed as a potential conflict of interest.
